# Covalent modification of iron oxide-poly(lithocholic acid) nanoparticles with folic acid or doxorubicin – an approach for enhanced cancer therapy†

**DOI:** 10.1039/d4ra08830a

**Published:** 2025-05-09

**Authors:** Dawid Szymczuk, Karolina H. Markiewicz, Katarzyna Niemirowicz-Laskowska, Diana Sawicka, Iwona Misztalewska-Turkowicz, Halina Car, Agnieszka Z. Wilczewska

**Affiliations:** a Faculty of Chemistry, University of Bialystok Ciolkowskiego 1K Bialystok 15-245 Poland agawilcz@uwb.edu.pl k.markiewicz@uwb.edu.pl; b Doctoral School of Exact and Natural Sciences, University of Bialystok Ciolkowskiego 1K Bialystok 15-245 Poland; c Department of Experimental Pharmacology, Medical University of Bialystok Szpitalna 37 Bialystok 15-295 Poland katarzyna.niemirowicz@umb.edu.pl; d Department of Clinical Pharmacology, Medical University of Bialystok Waszyngtona 15A Bialystok 15-274 Poland

## Abstract

This study explores the effectiveness against selected cancer cell lines of nano-engineered formulations composed of inorganic cores with steroid-based polymeric shells functionalized with either a targeting or chemotherapeutic agent. We present the synthesis and comprehensive characterization of iron oxide nanoparticles coated by polymeric layers derived from lithocholic acid with covalently affixed folic acid or doxorubicin entities. The cytotoxicity assessments against normal (RBCs, THP-1, CCD-1079sk, and H9C2(2-1) and cancerous (MCF-7, MDA-MB-231, and HeLa) cell lines were performed using two independent endpoint (MTT and neural red) assays. In the case of cancer cells, transepithelial electrical resistance (TERR) and caspase 8 and 9 expression were examined. Additionally, the impact on the activity of xenobiotic metabolizing enzymes from the cytochrome family has been assessed. The results of the study confirmed the selectivity of the synthesized hybrids against tested cancer cells and their ability to induce apoptosis *via* caspase activation.

## Abstract 2

This study explores the effectiveness against selected cancer cell lines of nano-engineered formulations composed of inorganic cores with steroid-based polymeric shells functionalized with either a targeting or chemotherapeutic agent.

## Graphical abstract

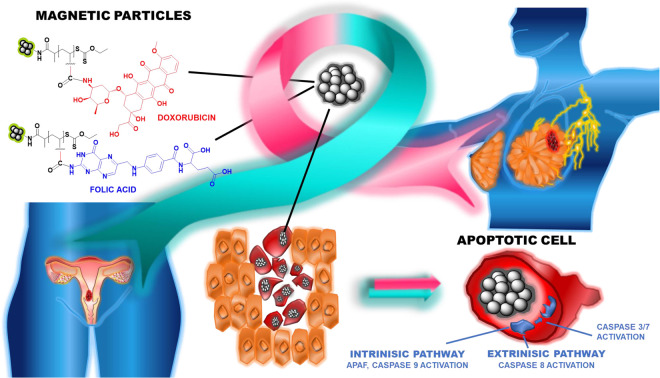

## Introduction

Developing smart drug delivery systems (SDDS) represents a valuable approach to cancer therapy. Combined or targeted therapy based on nanoparticles, polymers, or liposomes offers effective and groundbreaking results in cancer treatment compared to conventional therapy.^1–3^ These advanced systems aim to enhance the effectiveness of anticancer treatments while minimizing potential side effects.^4^

A promising cancer treatment strategy is therapy based on iron oxide nanoparticles (IONPs). Nanoparticles amplify the impact of cancer treatment by combining multiple therapeutic modalities, which can include sensitization techniques using small molecules and nanoparticles, or physical methodologies like radiation and phototherapy.^5,6^ The unique physicochemical properties of these nanoparticles, such as their small size and high and tuneable surface area, make them ideal carriers for anticancer drugs.^7,8^ Their biocompatibility and ability to modify their character with various ligands allow specific recognition and interaction with tumor cells.^9,10^ Furthermore, these nanohybrids' physicochemical characteristics offer solutions to several challenges in physicochemical and pharmacological aspects. These include increasing many anticancer drugs' aqueous solubility, targeted distribution within the body, and improving their biodegradability.^11^ It can overcome challenges such as drug resistance, a significant hurdle in cancer therapy, by concentrating drugs directly at the tumor site or sensitizing tumor cells to drug effect.^12^

Recent literature reports offer new possibilities for applying bile acids (BAs) in medicine.^13,14^ Lithoholic acid (LitA) exhibits antibacterial,^15^ antifungal,^16^ and antineoplastic activity toward colon, colorectal, and liver cancer cells.^17^ Recently, the potential of the lithocholic acid derivative as a drug delivery candidate targeting model lipid rafts was reported confirming its membrane-penetrating properties.^18^ In this view, introducing LitA as a component of nanocarrier for cancer treatment can improve the efficacy of carrier-drug conjugates, their biocompatibility and biodegradability. Furthermore, the presence of two reactive groups in lithocholic acid (carboxylic and hydroxyl) creates additional possibilities for modifying the carrier to improve its, *e.g.*, targeting properties.^19^

Folic acid (FA), also known as vitamin B9, plays a crucial role in nucleic acid synthesis and DNA repair. It is transported to healthy tissue cells through a specific cell-surface glycoprotein called the folate receptor. Folic acid is particularly valuable in cancer therapy, targeting tumor cells that overexpress folate receptors while sparing healthy cells.^20–22^ Recent studies have demonstrated that attaching folic acid to polymers can significantly improve the targeted delivery of anticancer drugs.^23,24^ The advantages of using folic acid in smart drug delivery systems are enhancing the specificity of drug delivery and increasing the therapeutic effect while reducing systemic toxicity.^25^ It allows for more precise dosing, improving the overall safety and efficacy of the treatment. Moreover, folic acid's biocompatibility and non-immunogenic nature make it an ideal choice for incorporation into various drug vehicles, including nanoparticles and liposomes.^26^ Research provided in our group indicated that the functionalization of aminosilane-coated magnetic nanoparticles by folic acid improves colorectal cancer therapy due to the elongated retention of MNPs and their ability to restrict tumor growth. Moreover, such functionalization protects against non-specific accumulation in the organs of healthy mice *in vivo*.^27^

The anthracycline group of antibiotics is notable for its broad antitumor and anticancer activity spectrum. Doxorubicin (DOX) stands as a pioneering and well-established chemotherapeutic agent that finds widespread application in the treatment of malignant tumors, including breast,^28^ lymphomas,^29^ sarcomas,^30^ bladder,^31^ ovarian,^32^ and stomach^33^ cancers. However, the highly toxic profile of the anthracyclines and the low selectivity for cancer cells lead to substantial dose-limiting acute and chronic toxicities. Doxorubicin is linked to significant harm to non-targeted tissues,^34^ including heart damage,^35,36^ myelosuppression,^37,38^ mucositis^39^ and nephrotoxicity.^40^ Recent data indicate an increasing prevalence of cardiovascular complications in breast cancer patients who have undergone DOX-based treatment. Furthermore, bone marrow suppression, thrombocytopenia, and doxorubicin-induced anemia are among the most important side effects of doxorubicin, which also significantly affects the dosage.^41,42^ Consequently, there is a considerable focus on minimizing the dosage of DOX to limit its adverse side effects.

To address the issues mentioned above and take advantage of the combination therapy, we propose new formulations based on iron oxide nanoparticles coated by polymeric layers derived from lithocholic acid (LitA) and covalently modified with folic acid (FA) or doxorubicin (DOX). FA/DOX was attached to polymer-magnetic hybrids by an amidation reaction between carboxylic acid and amine groups in FA/DOX. Several reports have highlighted the significant success of nano/micro-delivery systems featuring drug conjugates connected *via* amide linkages.^43^ The primary outcomes of these studies include enhanced antitumor effectiveness compared to the unbound drug, increased capacity for drug loading, prolonged duration of drug release, and a reduction in side effects relative to the bare drug.^44–48^ To determine the structure–activity relationship, we evaluated the cytotoxic effect and mode of action of the obtained polymer–inorganic hybrids in cancerous cell lines of cervical (HeLa) and breast cancer cells, which possess different molecular profiles (MCF-7 and MDA-MB-231). Furthermore, the selectivity and safety at the *in vitro* level were tested by applying the polymer–inorganic hybrids in normal cells (human red blood cells, monocytic cells, fibroblasts cells, and cardiomyocyte cells). Additionally, the influence of the materials on the activity of enzymes metabolizing xenobiotics, including cytochrome P450 CYP3A4 activity, was assessed.

## Experimental section

### Methods

#### Physicochemical characterization

Nuclear Magnetic Resonance (NMR) analyses were conducted using Bruker Avance II 400 and Avance DPX 200 instruments, operating at frequencies of 400 MHz for ^1^H NMR and 100 MHz for ^13^C NMR, with samples prepared in chloroform-d (CDCl_3_). Attenuated Total Reflectance Fourier Transform Infrared (ATR-FTIR) spectra were acquired using an ATR module on a Thermo Scientific Nicolet 6700 spectrometer. The spectral data were collected across a range of 4000 to 500 cm^−1^, aggregating 32 scans at a 4 cm^−1^ resolution. Thermal stability was evaluated using a Mettler Toledo Star system for Thermogravimetric Analysis/Differential Scanning Calorimetry (TGA/DSC), with 2–3 mg of the sample in alumina crucibles and a temperature ramp from 50 °C to 900 °C at 10 °C per minute under an argon atmosphere at 40 mL min^−1^. The formation of magnetic nanoparticles, their size and structure, was verified through Transmission Electron Microscopy (TEM) on a Tecnai G2 X-TWIN microscope. TEM samples were prepared on carbon-coated copper grids. Zeta potential and size distribution measurements were performed using Dynamic Light Scattering (DLS) on a Zetasizer Ultra (Malvern Panalytical Ltd, Malvern, UK), which features a 10 mW helium/neon laser at 633 nm wavelength. This analysis was automated by ZS XPLORER software (Malvern Panalytical Ltd, Malvern, UK) and conducted at 25 °C using a 173° backscatter detection setup. The particles were dispersed in water to a concentration of 0.25 mg mL^−1^, and the reported particle sizes represent the average hydrodynamic diameter from five separate measurements.

#### Biological studies

##### Hemocompatibility studies

In order to assess the efficacy of the tested agents in releasing hemoglobin from treated cells, fresh human red blood cells (RBCs) were obtained from healthy volunteers. The collected cells were suspended in phosphate-buffered saline (PBS) to establish a hematocrit of approximately 5%. The tested agents were prepared at a 0.1 mg mL^−1^ concentration and incubated for one hour at 37 °C. Following centrifugation, the relative hemoglobin concentration in the supernatants was spectrophotometrically assessed at a wavelength of 540 nm. The 0% hemolysis was obtained from samples following the addition of 10 μL of PBS, while the 100% hemolysis was obtained from samples in which 10 μL of Triton X-100 was added to disrupt all cell membranes.

##### Cells culture

The cytotoxicity of polymer–inorganic hybrids based on iron oxide nanoparticles comprising poly(lithocholic acid acrylate) or poly(acrylic acid) blocks and their modification by FA and DOX was determined against skin fibroblast CCD-1079sk, cardiomyocytes cells H9C2, breast cancer cells (MCF-7 and MDA-MB-231), and cervical cancer cells HeLa from American Type Culture Collection (Manassas, VA, USA). The cells were cultivated in 96-well plates at a density of 5–7 × 10^3^ cells per well until they reached full confluence in Eagle's Minimum Essential Medium-EMEM (ATCC) (for CCD-1079sk, HepG2, MCF-7, MDA-MB-231, HeLa), and in Dulbecco's modified Eagle's Medium-DMEM (ATCC) for H9c2(2-1) supplemented with 10% fetal bovine serum (FBS) (ATCC), 50 U mL^−1^ penicillin, and 50 mg mL^−1^ streptomycin (Gibco, Thermo Fisher Scientific, Inc., Waltham, MA, USA) under physiological conditions, at 37 °C with 5% CO_2_.

#### Cytotoxicity studies

In order to assess the cytotoxicity of the tested polymer–inorganic hybrids, a neutral red cell cytotoxicity assay was used. In brief, after 24 h incubation of cells with tested agents (0.1 mg mL^−1^), a neutral red solution (10 μL per well) was added and incubated for 2 h. Subsequently, the culturing medium was removed, and the cells were fixed for five minutes. Subsequently, the fixative solution was discarded, and the acidic solution was added to dissolve the dye. The absorbance was measured at a wavelength of 540 nm using a Varioscan Lux microplates reader (Termofisher). Values were described as a percent of control ± SD.

#### Cell metabolic activity

The metabolic activity of cells after the addition of the tested compounds was determined *via* the MTT assay method. After 24 h of incubation with tested agents (0.1 mg mL^−1^), the MTT assay protocol was followed. Subsequently, MTT reagent (5 mg mL^−1^) was added to each well and incubated for 3 h. The medium was removed from the wells, and 90 μL of DMSO (Alchem, Poland) was added with 10 μL of Sorensen's buffer (0.1 mol L^−1^ glycine with 0.1 mol L^−1^ NaCl equilibrated to pH 10.5). The absorbance was measured at a wavelength of 570 nm using a Varioscan Lux microplates reader (Termofisher). Values were described as a percent of control ± SD.

#### Measurement of the transepithelial electrical resistance (TEER)

The transepithelial electrical resistance (TEER) in treated cancerous cells MCF-7, MDA-MB-231, and HeLa was measured using the Evom Voltohmmeter supplemented with the End-Ohm chamber or STX2 chopstick (World Precision Instruments Inc., Sarasota, FL, USA) after 24 h incubation with tested agents added at concentration 0.25 mg mL^−1^.

#### Caspase immunoassay

The caspase 8 and 9 concentration in treated cells MCF-7, MDA-MB-231, and HeLa after 24 h incubation with tested agents and doxorubicin (positive control) was determined by Caspase Immunoassays (Promega). The agents were diluted in fresh media to the desired concentrations of 0.25 mg mL^−1^ and 50 μM for doxorubicin (DOX). Caspase immunoassay protocol was then performed. All results were performed in triplicate samples. The luminescence was measured using a Varioscan Lux microplates reader. The amount of luminescence displayed on the readout is proportional to the amount of caspase activity in the sample.

#### Cytochrome P450 activity

To examine the influence of the tested agents on the activity of enzyme metabolizing xenobiotics, a luminescence analysis of cytochrome P450 CYP3A4 activity was performed. Polymer–inorganic hybrids based on iron oxide nanoparticles were added to the HepG2 cells in concentrations of 0.1 and 0.25 mg mL^−1^ and incubated for 24 hours. The medium was replaced, and the same volume (50 μL) of fresh medium containing CYP3A4 substrate (3 μM) was added. After 1 hour of incubation, 25 μL of culture medium from each well was transferred at room temperature to a 96-well opaque white luminometric plate, and 25 μL of luciferin detection reagent was added. After 20 minutes, the luminescence was read.

#### Statistical analysis

Data were analyzed statistically by one-way analysis of variance (ANOVA). Tukey *post hoc* test was used to determine differences between groups. Statistical evaluation of results was performed using the GraphPadPrism software package.

## Results and discussion

### The formation, functionalization, and physicochemical characterization of polymer–inorganic hybrids

**Scheme 1:**
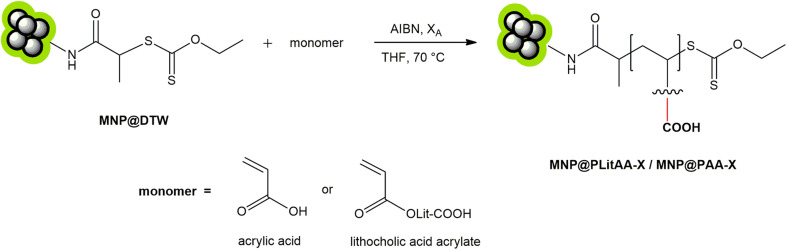
RAFT polymerization using iron oxide particles with immobilized chain transfer agent.

Polymeric shells were formed on dithiocarbonate-functionalized iron oxide nanoparticles (designated as MNP@X) through surface-initiated reversible addition-fragmentation chain transfer (RAFT) polymerization (see ESI†). The methodologies for synthesizing and physicochemical characterization of MNP@X nanoparticles have been detailed previously.^49–52^ In this study, MNP@X nanoparticles were utilized as chain transfer agents (CTAs) in the surface-initiated polymerization of synthesized lithocholic acid acrylate (LitAA) and acrylic acid (AA), resulting in the creation of two types of polymer-iron oxide hybrid materials (Scheme 1 and S1–S4†).

**Scheme 2:**
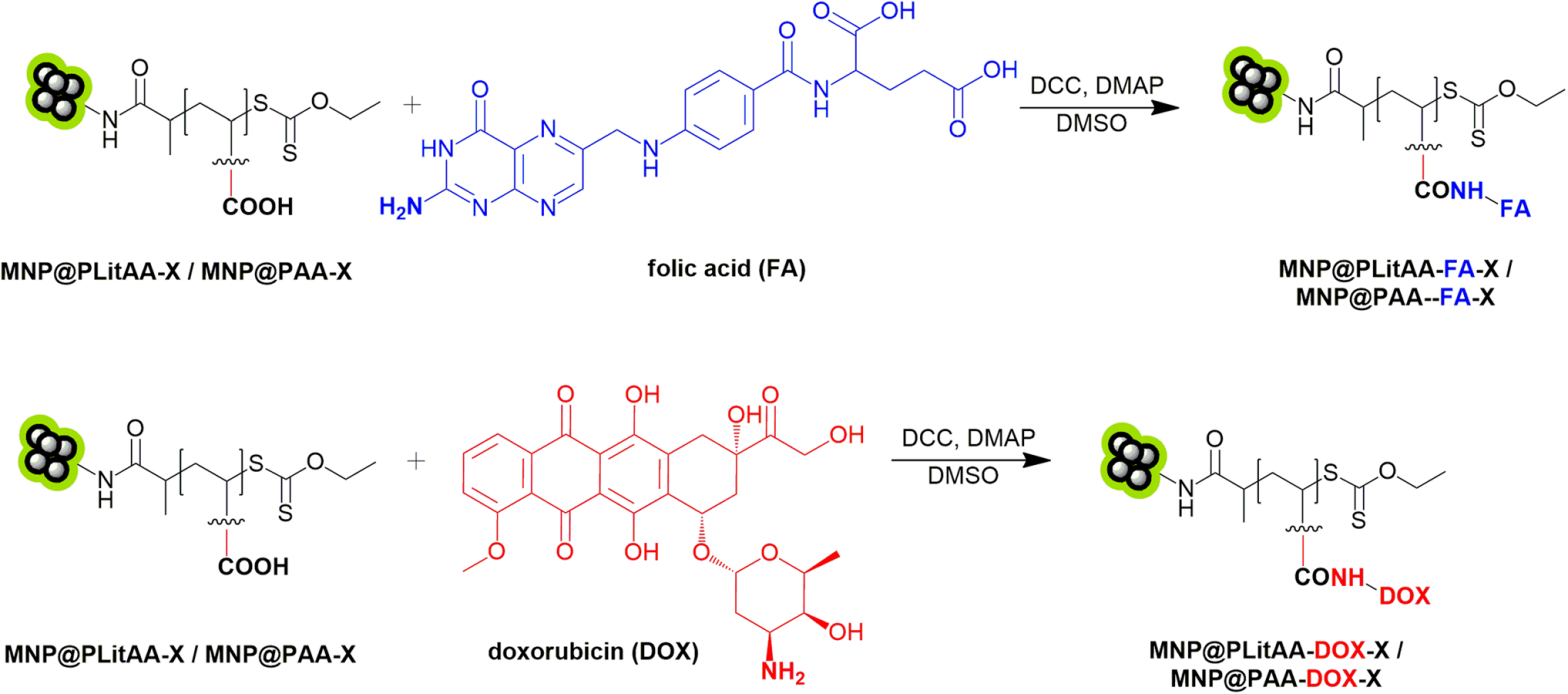
Modification of polymer–inorganic hybrids with biological active moieties.

PAA was selected for our research as a biocompatible and commercially available alternative to PLitAA with different accessibility of carboxyl groups within the polymer system. Subsequent functionalization of these hybrids with folic acid (FA) or doxorubicin (DOX) was achieved *via* amidation reactions (Scheme 2).

**Scheme 3:**
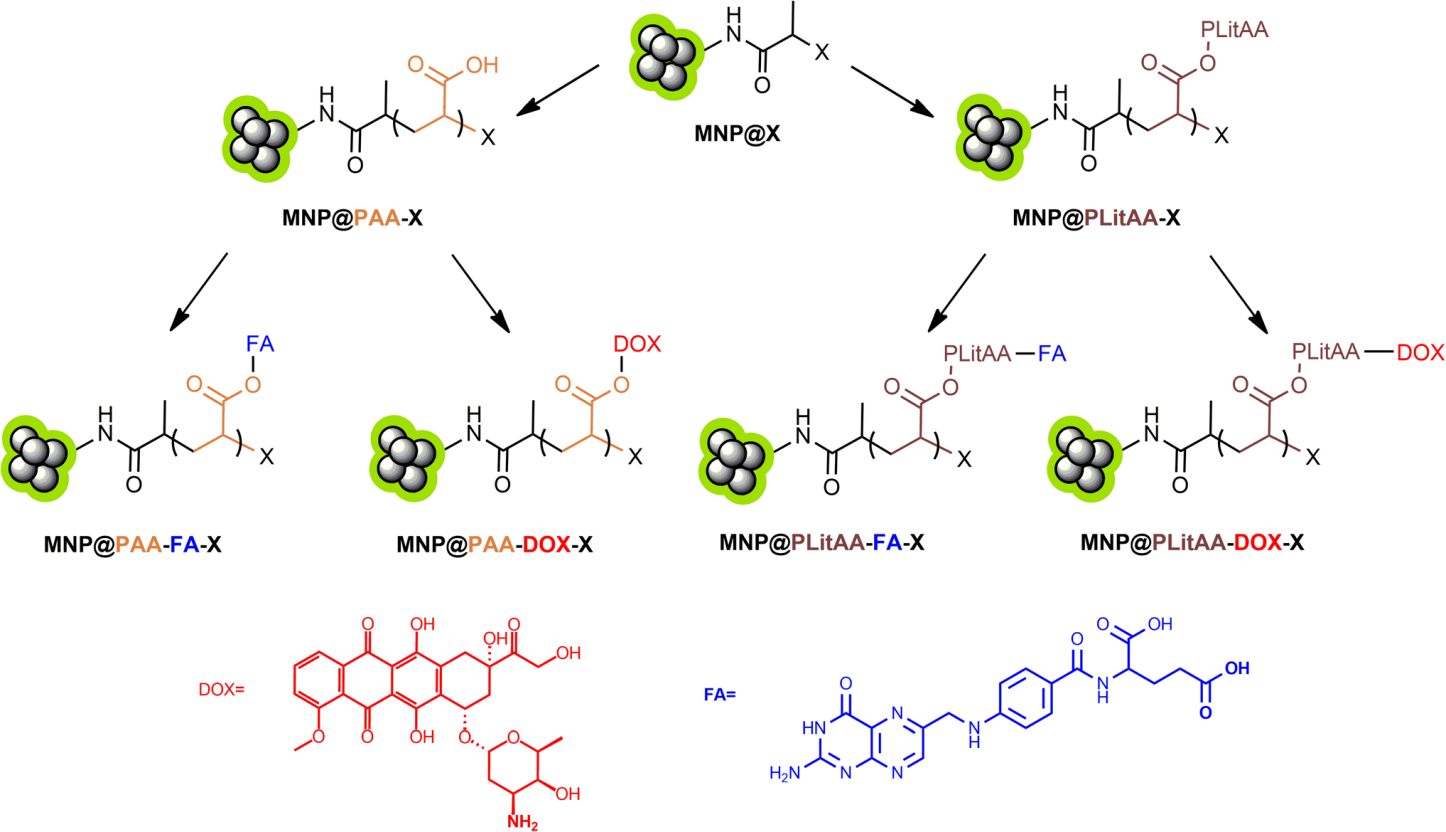
General presentation of obtained materials.

The amide bond plays a crucial role in the realm of drug delivery systems, particularly when it comes to the covalent conjugation of drugs to carriers. This chemical linkage is invaluable due to its stability and biocompatibility, essential attributes for the effective transportation and release of therapeutic agents within the human body. Amide bonds are resistant to premature cleavage in the circulatory system, ensuring the drug remains attached to its carrier until it reaches the targeted site. This stability is crucial for maintaining the drug's efficacy and minimizing off-target effects. Furthermore, the predictable hydrolytic degradation of amide bonds in specific physiological environments allows for the controlled release of the drug, enhancing its therapeutic efficacy while reducing side effects. The ability to fine-tune the hydrolysis rate of the amide bond enables the design of drug delivery systems that release their payload in response to the specific conditions of the target site, such as pH or the presence of certain enzymes. Consequently, the amide bond's stability, biocompatibility, and controlled degradability make it a pivotal element in developing efficient drug delivery platforms. The summary of all obtained materials is presented in Scheme 3.

**Fig. 1:**
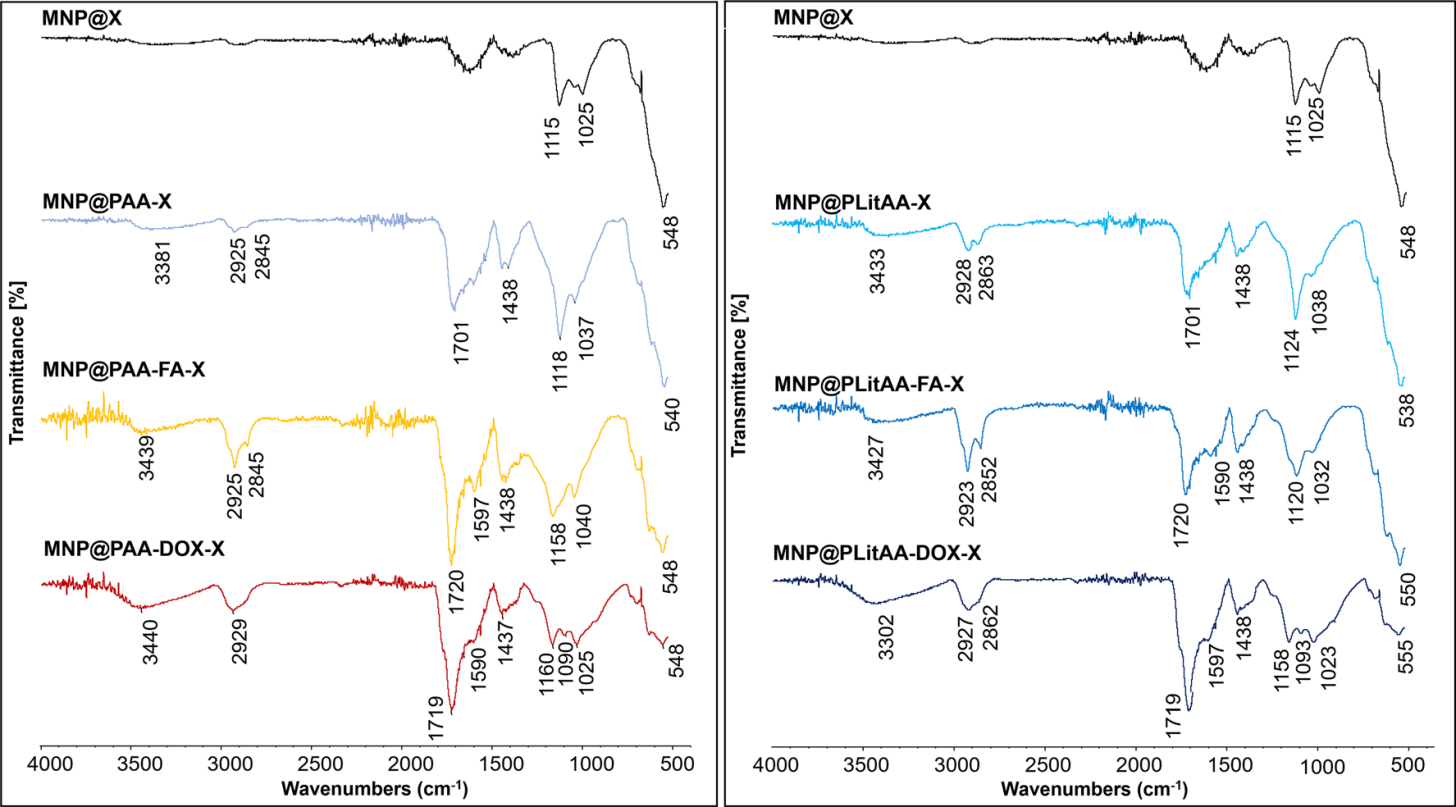
FT-IR spectra of obtained polymer–inorganic hybrids.

The ATR FT-IR spectra reveal changes in the characteristics of the hybrids compared to the starting material (MNP@X) (Fig. 1). For hybrids containing PAA and PLitAA, specific absorption bands show increased intensity, including the O–H stretching vibrations in carboxylic acids (3300 and 3000 cm^−1^), the C–H stretching vibrations in aliphatic chains (3000 to 2850 cm^−1^), the C=O stretching vibrations (1701 cm^−1^), and the O–H bending vibrations in carboxylic acids (1438 cm^−1^). In the FT-IR spectra of nanohybrids composed of PAA/PLitAA and FA/DOX, the presence of multiple functional groups leads to significant signal overlap, particularly in regions associated with carbonyl and amide groups. Nevertheless, analysis of these spectra suggests successful modification of the hybrids at each stage of synthesis. For nanohybrids incorporating FA, the spectra show increased intensities in the absorption bands for C–H stretching (3000–2850 cm^−1^) and a broad band with the maximum absorption at 1720 cm^−1^ due to carbonyl group stretching. This band might be related to carboxyl and lactam groups in FA-modified particles. It is also broadened due to the overlap of bands originating from the secondary amides vibrations (C=O and N–H), which appear around 1680–1515 cm^−1^. In the spectra of DOX-modified hybrids, patterns similar to those of FA-containing hybrids are observed. This includes changes in the intensity of the C–H stretching (3000–2850 cm^−1^) and a new signal from C=O stretching (from ketones and quinones of DOX) with maximum absorption at 1719 cm^−1^, which is broadened by the overlapping secondary amide bands (amide I and amide II). The signal at 1090 cm^−1^, which can be attributed to C–N stretching, is unique to the DOX-modified hybrids.

**Fig. 2:**
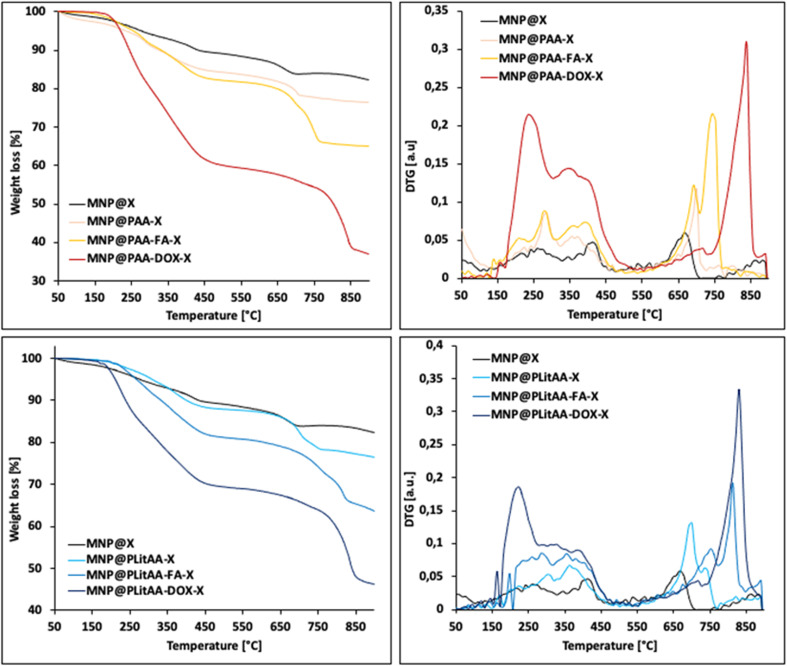
TG curves of polymer–inorganic hybrids.

Fig. 2 presents the results of the thermal analysis of the polymer–inorganic hybrid materials. Iron oxide cores are thermally stable across the entire temperature range used in the study, thus, the observed weight losses are due to the decomposition of the hybrid's organic coating layer. The initial material, MNP@X, exhibits a 16.2% weight loss, indicating the decomposition of its dithiocarbonate-modified amino-silica shell. This weight loss becomes more pronounced in all samples following polymerization, as shown in Table 1, suggesting the successful incorporation of polymers into the materials. Thermal degradation of these polymer-modified particles occurs within two specific temperature ranges: from 200 °C to 450 °C and from 450 °C to 750 °C. These ranges correspond to the decomposition of PAA/PLitAA and the amino-silica shell. The FA-modified hybrids show a decomposition pattern across a broader temperature span (100–800 °C), with five stages of weight loss at 125 °C, 265 °C, 420 °C, 650 °C, and 720 °C. These stages are associated with the decomposition of folic acid and the carbonization of the organic layer. Similarly, DOX-modified polymer–inorganic hybrids decompose between 150 °C and 800 °C in three sharp phases, occurring at 240 °C, 350 °C, and 800 °C. Notably, hybrids modified with biologically active substances exhibit a significantly higher weight loss than their unmodified counterparts, indicating a substantial change in composition and stability.

**Table 1:** Summary of physicochemical properties of polymer-modified MNP

Sample	Weight loss 804 °C [%]	Size [*d*, nm] H_2_O	PDI	*ζ* [mV] H_2_O
MNP@PLitAA	22	130 ± 4	0.09	−26
MNP@PLitAA-FA	30	152 ± 1	0.07	−28
MNP@PLitAA-DOX	43	275 ± 1	0.39	−28
MNP@PAA	23	163 ± 7	0.04	−33
MNP@PAA-FA	43	135 ± 3	0.03	−46
MNP@PAA-DOX	50	268 ± 7	0.14	−25

**Fig. 3:**
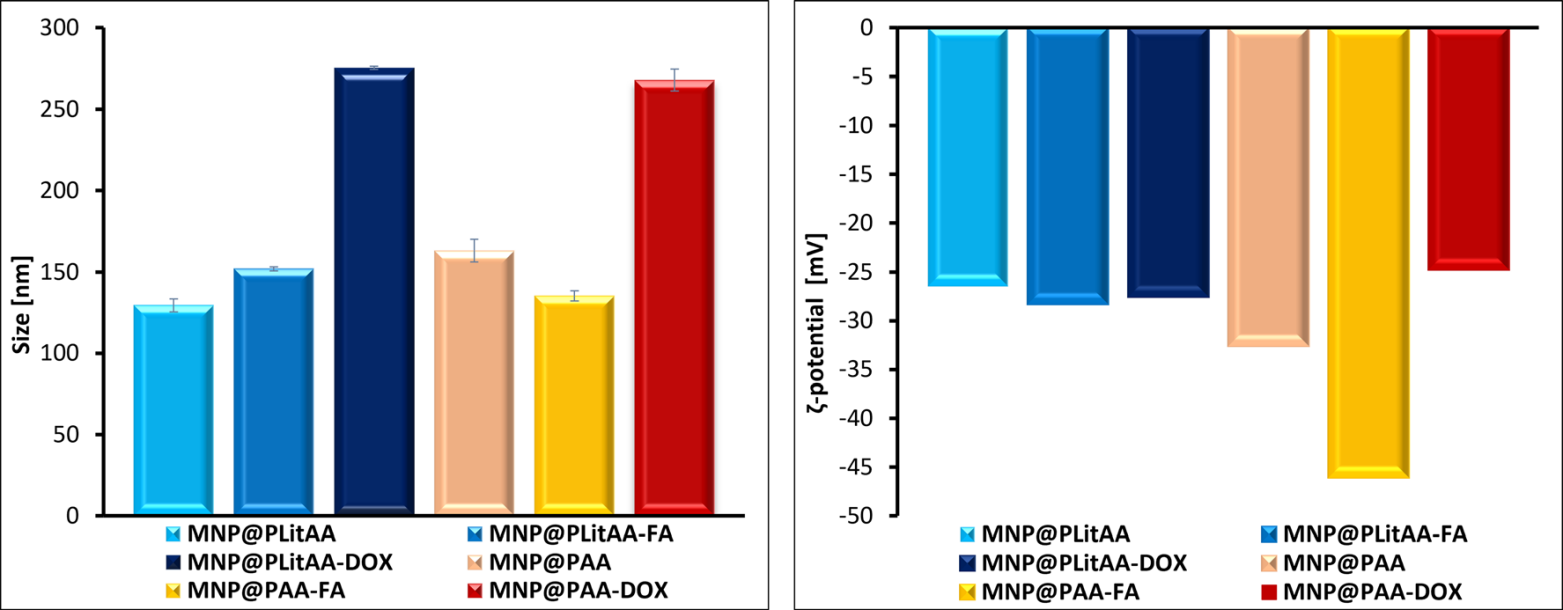
DLS and ELS results for polymer–inorganic hybrids.

Dynamic light scattering (DLS) measurements were performed to analyze the size distribution of particles in water. The starting material, MNP@NH_2_, exhibited a hydrodynamic diameter of around 160 nanometers. Polymer–inorganic hybrids and their modifications with biologically active substances revealed sizes within the range of 130 to 270 nm (Fig. 3 and Table 1). In general, an increase in shell content led to an increase in particle size. Modifications of the hybrids with doxorubicin resulted in a significant increase in the hydrodynamic diameter of the particles, which is in line with TG analysis results.

Electrophoretic light scattering (*ζ*-potential) measurements were conducted to evaluate the stability of these systems in aqueous environments, as depicted in Fig. 3. The starting material, MNP@NH_2_, exhibited a *ζ*-potential of 12 mV. After polymerization, the *ζ*-potential significantly decreased, reaching −26 mV for MNP@PLitAA and −32 mV for MNP@PAA, suggesting improved stability. Introducing folic acid into these systems further lowered the *ζ*-potential values, enhancing their stability in water. Conversely, the modification of the particle surface with DOX did not significantly affect the *ζ*-potential values compared to the unmodified material.

Moreover, to evaluate the colloidal stability of polymer-modified iron oxide nanoparticle suspensions, their zeta potential and particle size distribution were analyzed over time. The particles were dispersed in distilled water and characterized immediately after dispersion (*t* = 0), after 24 hours, and after 7 days of storage at room temperature.

Immediately after dispersion, the suspensions exhibited high homogeneity and a stable zeta potential ranging from −25 mV to −46 mV, indicating good electrostatic stability. After 24 hours, the properties of the suspensions – including particle size distribution and zeta potential – remained unchanged compared to the freshly prepared samples, confirming short-term colloidal stability. After 7 days of storage, partial sedimentation of the particles was observed, indicating a decrease in the stability of the suspensions over time. However, the use of ultrasonic redispersion effectively restored the original properties of the systems, both in terms of particle size distribution and zeta potential. The resulting suspensions were once again homogeneous and stable, confirming that the aggregation process was reversible. Therefore, to ensure the highest quality and reproducibility, the nanoparticle suspensions intended for biological experiments were prepared directly prior to use.

The TEM images (Fig. S4†) present MNP@PLitAA particles under two different magnification levels, displaying spherical iron oxide particles with 10 to 15 nm diameters. These particles cluster together to form aggregates ranging from 100 to 280 nm. The images depict the iron oxide magnetic cores as dark spots surrounded by a lighter, thin polymer shell, indicating the core–shell structure of the particles. This visualization highlights the successful modification of iron oxide particles by the polymer.

The summary of types and physicochemical properties of obtained hybrids are presented in Table 1.

### Biological activity

**Fig. 4:**
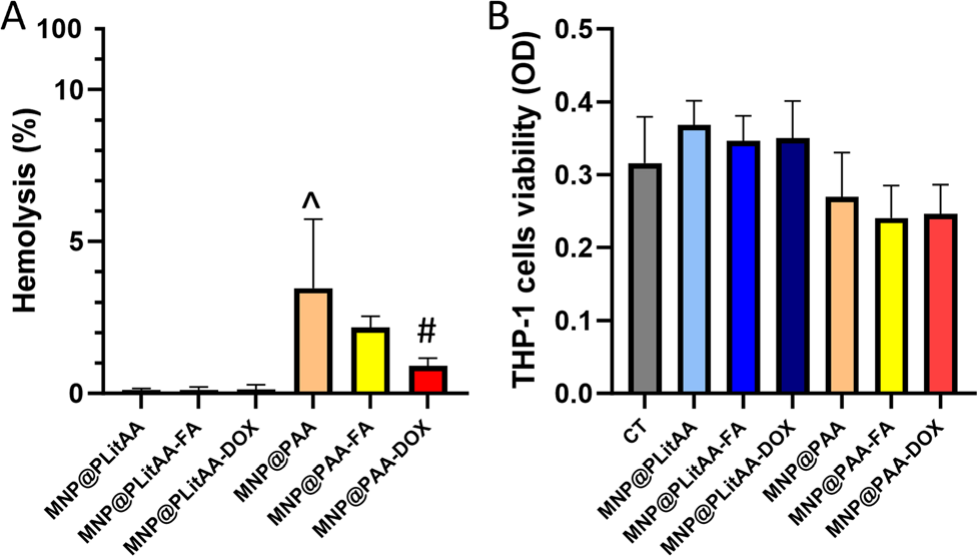
Hemocompatibility of polymer–inorganic hybrids. Lack of hemolytic activity (panel A) and viability of human monocytic cells (panel B) after 24 h incubation with polymer–inorganic hybrids (0.1 mg mL^−1^). Statistical significance: *vs.* control was marked (*); PLitAA *vs.* PAA was marked (^); *vs.* DOX moiety (#) *p* ≤ 0.05. The data presented constitute average results from three measurements ± SD.

The compatibility with host cells strongly determines the successful further application of nanoparticles. It is established that lack of homogenicity in the nanoparticles population might cause several problems, such as hemolysis or general toxicity. In effect, before *in vivo* application, determination of the hemolytic activity of MNPs is recommended. For this purpose, the hemolysis assay was performed to test the hemolytic activity of the synthesized carriers. The results showed (Fig. 4) that all tested hybrids are compatible with RBCs (hemolysis below 5%), which is in agreement with the ASTM F756 standard that has been used to determine the hemolytic activity (% hemolysis) of different nanomaterials.^53^ Interestingly, statistical significance was noted between MNP@PLitAA and MNP@PAA. MNP@PAA and their FA-derivatives can be classified as slightly hemolytic – the percentage of hemolysis is 2–5%. However, these findings do not exclude using this nanomaterial in magnetic hyperthermia procedures for cancer treatment.^54^

Monocytes are known as one of the most potent innate immune cells due to fast activation upon receiving an inflammatory signal. They originate in the bone marrow and circulate within the bloodstream, comprising 2–10% of the white blood cell population.^55^ It is established that chemotherapy affects non-target cells, including those involved in the immune response. DOX-based therapy causes toxicity to the blood progenitors and changes in cell markers, resulting in altering some immunological parameters. Studies by Lower *et al.* suggested that chemotherapy causes a reversible impairment of monocyte function.^56^ Moreover, the major side effect of long-term DOX-based chemotherapy is monocytopenia.^57^ In this study, the evaluation of whether the doxorubicin attached to the surface of magnetic nanohybrid affects the viability and metabolic activity of human monocytes was performed. For this purpose, an MTS assay was engaged, which is based on estimating metabolic activity that correlates with their viability. Results presented in Fig. 4 indicated that all tested compounds are characterized by a lack of toxicity against treated THP-1 cells.

To evaluate possible cytotoxic effects in noncancerous cells, fibroblasts CCD-1079Sk and cardiomyocyte cells H9c2(2-1) were exposed for 24 h to polymer–inorganic hybrids. Agents were applied at a concentration of 0.1 mg mL^−1^ per well and evaluated in terms of proliferation (MTT test) and viability of cell (neutral red assay).^58^ The MTT assay is used to quantify the cell metabolic activity by colorimetry because metabolically active cells possess the ability to transform a water-soluble dye [3-(4,5-dimethylthiazol-2-yl)-2,5-diphenyltetrazolium bromide] into an insoluble formazan. In turn, neutral red is a eurhodin dye that specifically stains lysosomes in viable cells. The core principle of this assay relies on detecting viable cells through the uptake of the dye-neutral red. Viable cells can actively transport neutral red into their lysosomes, where it is incorporated into the cell's internal membranes. In contrast, nonviable cells are unable to take up this chromophore.

Fibroblasts are cells that contribute to the formation of connective tissue by secreting collagen proteins that help maintain the structural framework of tissues.^59^ It is established that during DOX-chemotherapy, this physiological process could be significantly disrupted.^60^ It is also established that the highest risk of cardiac incidence and complications is observed in patients who have received anthracyclines.^61^ However, the toxic effects on the myocardium of this group of drugs represent a significant limiting factor in the full utilization of anthracyclines in anticancer therapy. The mechanism of cardiotoxicity induced by anthracyclines differs from their mechanism of action as anticancer agents. The anticancer effect of anthracyclines is primarily due to the binding of these drugs to DNA, which damages nucleic acids. Cardiotoxicity is a consequence of the sequence of disruption of mitochondrial function and structure.

**Fig. 5:**
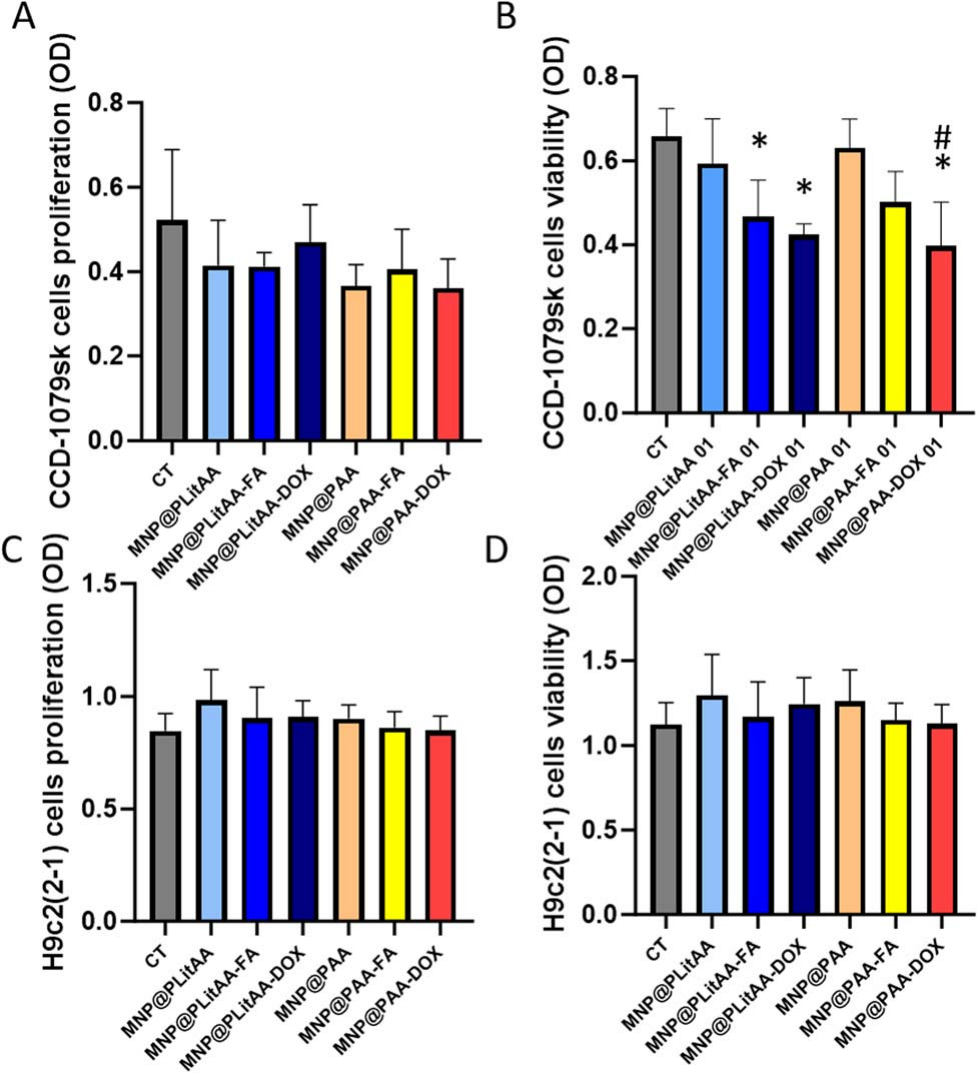
Compatibility of polymer–inorganic hybrids against normal cells. Proliferation (panel A) and viability (panel B) of human fibroblast cells after 24 h incubation with polymer–inorganic hybrids (0.1 mg mL^−1^). Proliferation (panel C) and viability (panel D) of cardiomyocyte cells after 24 h incubation with polymer–inorganic hybrids (0.1 mg mL^−1^). Statistical significance: *vs.* control was marked (*); PLitAA *vs.* PAA was marked (^); *vs.* DOX moiety (#) *p* ≤ 0.05. The data presented constitute average results from three measurements ± SD.

In this study, representatives of normal cells, fibroblast CCD-1079Sk cells, and cardiomyocyte H9c2(2-1) cells were exposed for 24 h to polymer–inorganic hybrids. Agents were applied at a concentration of 0.1 mg per mL per well and evaluated in terms of proliferation and viability of the cell. Our results indicated (Fig. 5) that tested carriers had no toxic impact on CCD-1079Sk cells' as well as H9c2 (2-1) cells' metabolic activity if applied at a concentration of 0.1 mg mL^−1^. In another set of experiments, where a neutral red assay was used, the DOX decorated hybrid caused a statistically significant effect, showing that mortality was not bigger than 20% in the case of CCD-1079Sk cells compared to untreated cells. However, based on data published by López-García *et al.*, ISO 10993-5 recommendation classified a 20% decrease in the number of viable cells as non-toxic. In effect, it could be concluded that fibroblast and cardiomyocyte cells subjected to tested polymer–inorganic hybrids exert good viability and metabolic activity, which correlate with the ability of proliferation and cell division. It could be associated with the fact that normal cells, including fibroblasts, possess specific enzymatic systems such as heme-oxygenase-1 and metallothioneins, which regulate oxidative stress induced by nanoparticles and provide detoxification.^62^ Moreover, compared to cancer cells, in normal cells, there are differences in plasma-membrane architecture (lipids composition surface charge and receptors) that prevent the nanoparticles' internalization.^63^

**Fig. 6:**
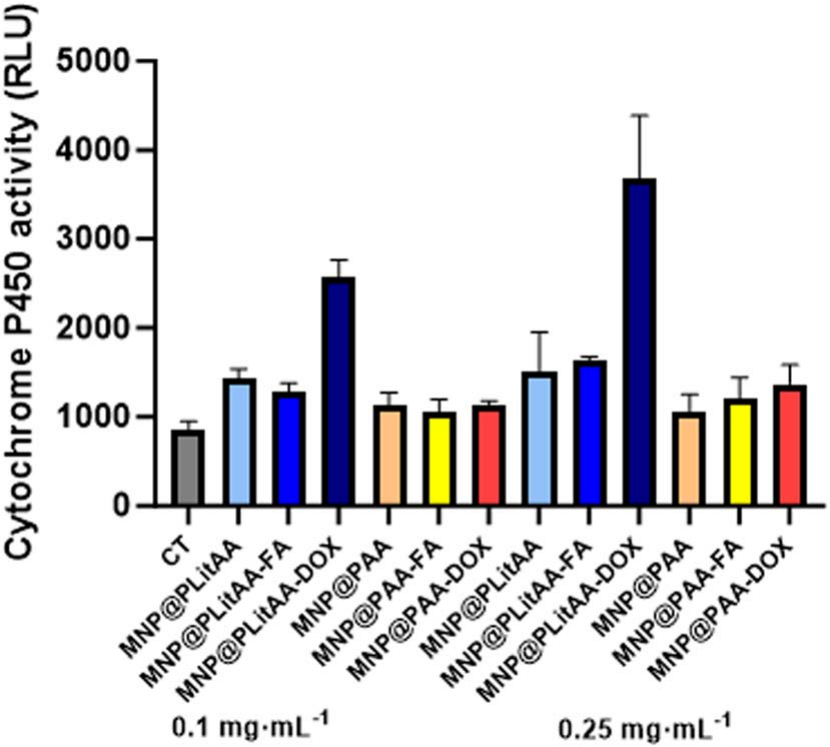
Changes in the activity of cytochrome P450 of human hepatoma cells after incubation with polymer–inorganic hybrids applied at concentrations 0.1 and 0.25 mg mL^−1^. Statistical significance: *vs.* control was marked (*); PLitAA *vs.* PAA was marked (^); *vs.* DOX moiety (#) *p* ≤ 0.05; dose-depend effect ($). The data presented constitute average results from three measurements ± SD.

To date, there are a lot of available reports about toxicity studies on nanoparticles; however, work on pharmacokinetics, especially metabolism, is limited.^64^ The liver is a crucial organ with complex functions. It maintains homeostasis by regulating metabolism and neutralizing and removing products and metabolites such as xenobiotics, drugs, and toxins from the body.^65^ Hence, to assess how polymeric/magnetic hybrid nanoparticles influence metabolic steps, HepG2 cells as a cell-based model in terms of screening for CYP inducers/inhibitors have been engaged.^66^ The cell line mentioned above has maintained many of the metabolic functions of normal hepatocytes; therefore, it is a useful cellular model to study the toxic effects of xenobiotics, including nanoparticles, at the *in vitro* level.^67^ Results presented in Fig. 6 indicated that a statistically significant increase in the activity of CYP3A4 was noted after incubation of cells with MNP@PLitAA derivatives in both tested concentrations. A marked rise in enzyme activity was also indicated if DOX molecules were attached. Interestingly, in the case of DOX-decorated hybrids containing litocholic acid, concentration-dependent increased activity of xenobiotic metabolizing enzymes was observed. These results align with previously published reports, where authors confirmed that DOX alters the expression of several P450. They indicated it caused the induction of CYP1B1 and CYP4A enzymes in the liver and kidney of rats.^68^ The abovementioned could be associated with the fact that CYP3A4 is mainly responsible for the phase I metabolism of doxorubicin.^69^ The cytochrome family plays a significant role in the detoxification of xenobiotics, cellular metabolism, and homeostasis. Identifying the impact on CYP enzyme activity, including induction or inhibition, is a major mechanism underlying drug–drug interactions. These results are important for further drug interaction prediction.^70^

In the final step, the anticancer potential of polymer-inorganic hybrids was evaluated by a battery of tests such as neutral red and MTT assay, measuring the Trans-Epithelial Electrical Resistance (TEER), and caspase 8 and 9 activity in breast (MCF-7 and MDA-MB-231) and cervical (HeLa) cancer models.

**Fig. 7:**
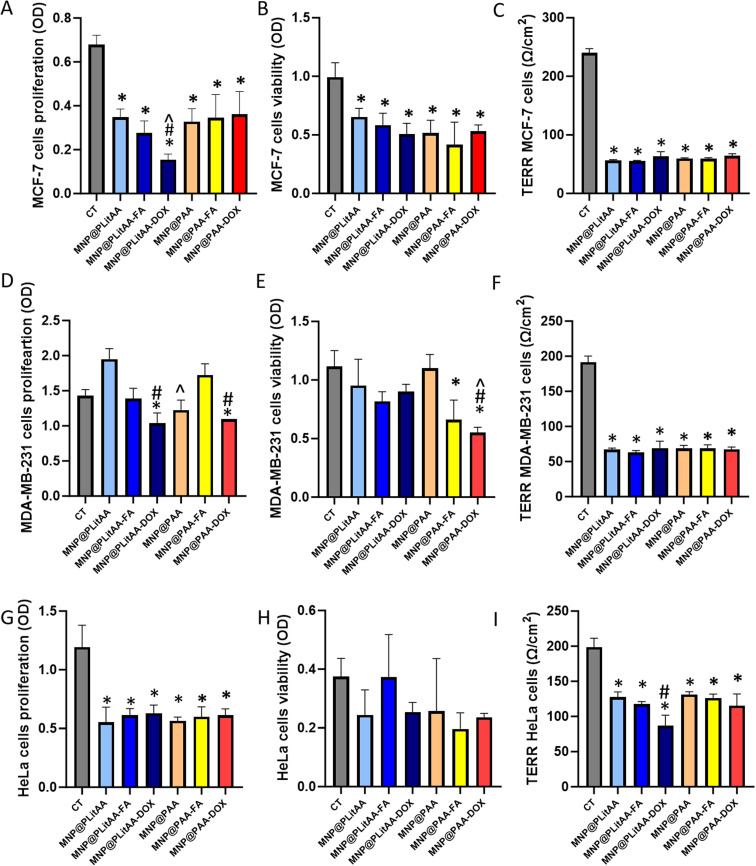
Anticancer activity of polymer–inorganic hybrids. Proliferation (A, D and G), viability (B, E and H) and TERR (C, F and I) MCF-7, MDA-MB-231 and HeLa cells after 24 h treatment by polymer–inorganic hybrids applied at concentration 0.1 mg mL^−1^ (proliferation and viability) and 0.25 mg mL^−1^ for TERR analysis. Statistical significance: *vs.* control was marked (*); PLitAA *vs.* PAA was marked (^); *vs.* DOX moiety (#) *p* ≤ 0.05. The data presented constitute average results from three measurements ± SD.

MCF-7 cells are derived from a human mammary adenocarcinoma and are known as a noninvasive and hormone-responding cell line. The cells mentioned above possess positive expression of estrogen, progesterone, and glucocorticoid receptors.^71^ As shown in Fig. 7, all tested agents can decrease the proliferation and viability of treated cells. Interestingly, carriers comprising PLitAA exert stronger antiproliferative activity than the ones containing PAA. The presence of DOX in the PLitAA structure significantly increases the cytotoxic effect compared to unfunctionalized carriers. Obtained data has also been confirmed by TERR measurement, where a significant decrease in resistance directly associated with the decrease of monolayer barrier integrity was found if compared to control. Next, to assess whether the molecular profile of cells had an impact on the displayed cytotoxic effect, another type of breast cancer cell line – MDA-MB-231 was employed. This cell line is frequently evaluated as a model of late-stage breast cancer and is characterized as epithelial adenocarcinoma, ER-negative, poorly differentiated, and highly tumorigenic.^72^ Results have shown that the aforementioned cells were more resistant to treatment by our nanosystems. A statistically marked decrease in proliferation and viability was only observed after treatment by hybrids decorated by DOX molecules. Analysis of the mortality percentage based on data from neutral red assay revealed a significantly better cytotoxic effect in the case of carriers functionalized by DOX with PAA shells than those composed of PLitAA. However, increasing the concentration to 0.25 mg mL^−1^ caused that all hybrids, regardless of their structure, alter the TEER after 24 h of exposure. In another set of experiments, representatives of cervical cancer cells were exposed to synthesized hybrids. The results from the proliferation assay exhibited that all agents can alter cell division. The cell viability was recorded as 70–50%, which indicated weak to marked cytotoxicity, while the maximum decrease in cell viability was measured for DOX-decorated ones. Data from TERR measurement demonstrated a significant decrease in resistance depending on the presence of DOX in the PLitAA-containing hybrid.

**Fig. 8:**
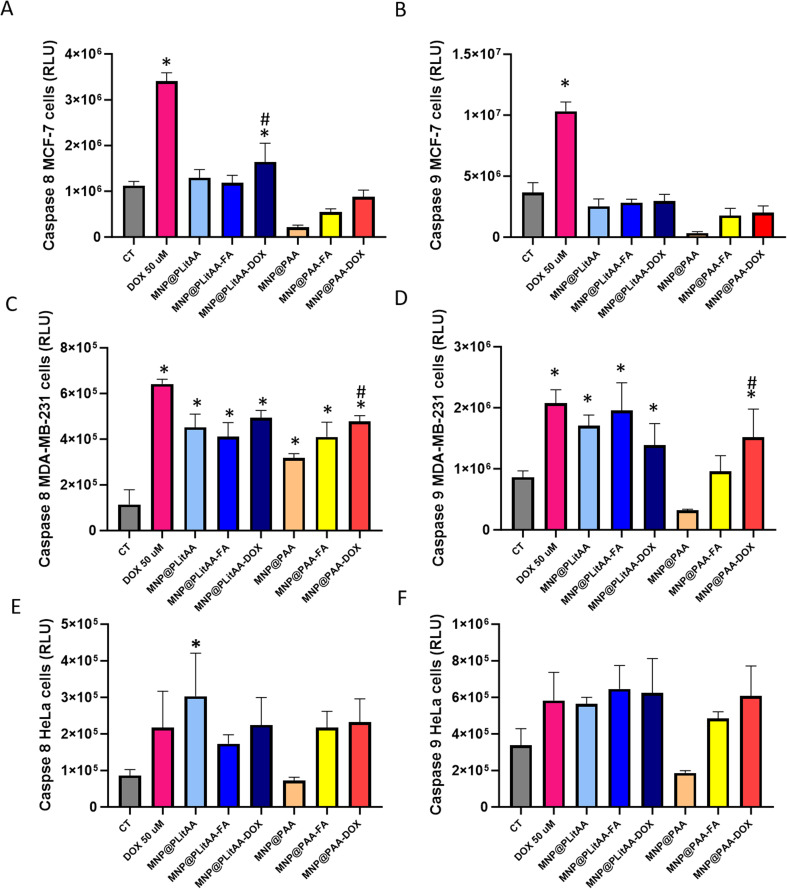
Caspase 8 and 9 activity in breast (MCF-7, MDA-MB-231) and cervical (HeLa) cell lines after 24 h treatment by polymer–inorganic hybrids. Caspase 8 (A, C and E) and caspase 9 (B, D and F) in MCF-7, MDA-MB-231, and HeLa cells after 24 h treatment by polymer–inorganic hybrids applied at concentration 0.25 mg mL^−1^. Statistical significance: *vs.* control was marked (*); PLitAA *vs.* PAA was marked (^); *vs.* DOX moiety (#) *p* ≤ 0.05. The data presented constitute average results from three measurements ± SD.

It is established that anticancer drugs induce apoptosis, which plays a pivotal role in cancer drug development. For this reason, we tested the ability of the synthesized hybrids to induce controlled cell death *via* caspase activation pathways. MCF-7, MDA-MB-231, and HeLa cells were exposed to nanosystems applied at a concentration of 0.25 mg mL^−1^ for 24 h. DOX at a concentration of 50 μM was used as a positive control. Caspase 8 is a cysteine protease that initiates apoptosis *via* the extrinsic pathway and has an impact on necroptosis and gene regulation of cell adhesion and/or migration.^73^ Caspase 9 activation is associated with the mitochondrial or intrinsic pathway.^74^ The results obtained from the induction of apoptosis *via* activation of caspase 8 are shown in Fig. 8. A statistically significant induction in caspase 8 was measured in MCF-7 cells exposed to DOX-decorated PLitAA-based hybrids. In the case of MDA-MB-231, a significant increase of caspase-8 was observed after incubating treated cells with all synthesized nanocarriers. The 24 hour exposition of cervical cancer cells to nanohybrids also markedly increased the caspase 8 activation, especially in the cases of DOX-functionalized ones. Analysis of caspase-9 level showed no significant increase when synthesized hybrids were incubated with the MCF-7 cells. In turn, a marked increase of caspase-9 was observed in MDA-MB-231 cells exposed to MNP@PLitAA-based carriers and DOX-decorated PAA ones. Similar results were obtained for HeLa cells. A 2-fold increase – but non-statistically significant – in caspase-9 level after incubation with PLitAA-based carriers and DOX-decorated ones was noted.

## Conclusions

Herein, we presented new formulations of iron oxide nanoparticles covered with lithocholic acid-based shells with covalently attached folic acid or doxorubicin molecules for enhanced cancer therapy. Cytotoxicity was evaluated in normal (RBCs, THP-1, CCD-1079sk, and H9c2(2-1)) and cancerous (MCF-7, MDA-MB-231, and HeLa) cell lines using MTT and Neutral Red assays. For cancer cells, transepithelial electrical resistance (TEER) and the expression of caspases 8 and 9 were analyzed. Additionally, the impact on the activity of cytochrome family xenobiotic metabolizing enzymes was assessed. Our results have shown that evaluated nanohybrids represent a promising class of nanocarriers. Cytotoxicity assessments of synthesized nanohybrids indicated good compatibility with normal cells and high cytotoxic efficacy against tested cancerous cells with the ability to induce apoptosis *via* caspase-depend activation.

## Data availability

The data supporting this article have been included as part of the ESI.†

## Author contributions

All the authors contributed substantially to this paper. Conceptualization, K. H. M., A. Z. W., and K. N.-L.; methodology, K. H. M., A. Z. W., and K. N.-L., D. S.; investigation, D. Sz., A. S., K. N.-L., K. H. M. I. M.-T.; writing—original draft preparation, D. Sz., K. H. M., K. N.-L., and D. S.; writing—review and editing, A. Z. W. and H. C.; visualization, D. Sz.; K. N.-L., and A. Z. W.; project administration, A. Z. W.; funding acquisition, A. Z. W., K. N.-L., H. C. All authors have read and agreed to the published version of the manuscript.

## Conflicts of interest

There are no conflicts to declare.

## Supplementary Material

RA-015-D4RA08830A-s001
