# Supplementary material for: Covalent modification of iron oxide-poly(lithocholic acid) nanoparticles with folic acid or doxorubicin – an approach for enhanced cancer therapy
Source: RSC Adv. 2025 May 9;15(18):14246–58. doi: 10.1039/d4ra08830a (PMC12063073; doi:10.1039/d4ra08830a)
Supplement: RA-015-D4RA08830A-s001 [file RA-015-D4RA08830A-s001.pdf]

Supplementary Information (SI) for RSC Advances.
This journal is © The Royal Society of Chemistry 2025

Covalent modification of iron oxide-poly(lithocholic acid) nanoparticles with folic acid or doxorubicin - an approach for enhanced cancer therapy

Dawid Szymczuk[1,2], Karolina H. Markiewicz[1], Katarzyna Niemirowicz-Laskowska[3]*, Diana Sawicka[3], Iwona Misztalewska-Turkowicz,[1] Halina Car[3,4], Agnieszka Z. Wilczewska[1]*

1. Faculty of Chemistry, University of Bialystok, Ciolkowskiego 1K, 15-245 Bialystok, Poland, k.markiewicz@uwb.edu.pl, agawilcz@uwb.edu.pl.
2. Doctoral School of Exact and Natural Sciences, University of Bialystok, Ciolkowskiego 1K, 15-245 Bialystok, Poland.
3. Department of Experimental Pharmacology, Medical University of Bialystok, Szpitalna 37, 15-295 Bialystok, Poland, katarzyna.niemirowicz@umb.edu.pl.
4. Department of Clinical Pharmacology, Medical University of Bialystok, Waszyngtona 15A, 15-274 Bialystok, Poland.

*agawilcz@uwb.edu.pl, katarzyna.niemirowicz-laskowska@umb.edu.pl

Keywords: magnetic particles, lithocholic acid, folic acid, doxorubicin, breast cancer, covalent drug bonding, combined therapy

Table of content:

Figure S1. ^{1}H NMR spectrum of LitAA monomer.

Figure S2. FT-IR spectrum of MNP@PAA-DOX.

Figure S3. FT-IR spectrum of MNP@PLitAA-DOX.

Figure S4. DLS size distribution and corellogram of MNP@PLitAA.

Figure S4. DLS size distribution and corellogram of MNP@PAA.

Figure S4. TEM images of MNP@PLitAA.

Experimental section

Materials

Iron(III) chloride hexahydrate $FeCl_3·6H_2O$ (Sigma-Aldrich), iron(II) chloride tetrahydrate $FeCl_2·6H_2O$ (Sigma-Aldrich), 3-aminopropyltrimethoxysilane (APTMS, 97 %, Sigma-Aldrich), 2-bromopropionyl bromide (97%, Sigma-Aldrich), carbon disulfide (CS_2, ≥99.9 %, Sigma-Aldrich), potassium hydroxide (KOH, Pure, Avantor), Lithocholic acid (95%, Alfa Aesar), triethylamine (Et_3N, Avantor), acrylic acid (96%, Sigma-Aldrich), *N,N'*-dicyclohexylcarbodiimide (DCC, 99%, Merck), 4-dimethylaminopyridine (DMAP, ≥99 %, Sigma-Aldrich), doxorubicin hydrochloride (DOX, AmBeeD, USA), folic acid (FA, 95-102%, Alfa Aesar), acryloyl chloride (AcrCl, ≥ 97%,

Aldrich) were used as received. The initiator, 2,2′-azobis(2-methylpropionitrile) (AIBN, ≥98%, Merck), was recrystallized from methanol. O-ethyl-S-(1-methoxycarbonyl)ethyldithio carbonate - X_A was synthesized according to the well-known procedure.[1] All organic solvents were purchased from Avantor Performance Materials, Poland S.A., and were distilled before use. Deuterated chloroform ($CDCl_3$) was purchased from Euroistop. Glassware was dried in a laboratory oven at 110 °C for all experiments.

Synthetic procedures

LitAA (lithocholic acid based monomer): (4R)-4-((3R,8R,9S,10S,13R,14S,17R)-3-(acryloyloxy)-10,13-dimethylhexadecahydro-1H-cyclopenta[a]phenanthren-17-yl)pentanoic acid: Lithocholic acid (10 g, 26.6 mmol), anhydrous dichloromethane (700 mL), and triethylamine (4.04 g, 39.9 mmol) were placed in a flask. The mixture was placed on a magnetic stirrer in an ice bath (0 °C) under an inert argon flow and protected from light. Then acryloyl chloride (3.13 g, 34.6 mmol) was added dropwise. After 1h of stirring, the mixture was washed with 5% hydrochloric acid and distilled water. The organic layer was dried over magnesium sulfate and evaporated (protected from light, at 40 °C). The final compound was purified by column chromatography (hexane-ethyl acetate (8:2)). The product was dried using an oil pump. 4.14 g of white powder was obtained. **^{1}H NMR** δ_H (400Hz, $CDCl_3$): 6.33 (dd, J = 17.2 Hz, 1H), 5,85 (dd, J = 10.2 Hz, 1H), 4.35 (m, 1H), 0.96 (m, 3H), 0.70 (s, 3H).

MNP@PAA: Dithiocarbonate-functionalized iron oxide particles (MNP@X) were obtained according to the reported procedure.[2,3] A 10 mL suspension of MNP@X in ethanol underwent magnetic separation. The particles were washed multiple times with THF. 100 mg of MNP@X, 200 mg of acrylic acid (0.190 mL, 2.78 mmol), 0.052 mg of *O*-ethyl-*S*-(1-methoxycarbonyl)ethyldithiocarbonate - X_A (0.0025 mmol), and 4 mL of anhydrous tetrahydrofuran were combined in a test tube, sonicated, and purged with argon gas for half an hour. Then, 8 mg of AIBN (0.048 mmol) was introduced in two separate portions (at the start and after two hours of the reaction), and the mixture was subsequently maintained at a temperature of 70 °C for four hours. The polymerization process was stopped by diluting the reaction mixture with THF and magnetic decantation. This process of redispersing and separating the mixture was executed 5 times. The particles were dispersed and stored in 50 mL THF (~2.2 mg·mL^{-1}). **MNP@PAA** FT IR (ν_{max}, cm^{-1}): 3500-3000 (O-H), 2925 (C-H), 2845 (C-H), 1701 (C=O), 1438 (O-H), 1118(C-N), 10367(Si-O-Si), 540 (Fe-O).

MNP@PLitAA: A 10 mL suspension of MNP@X in ethanol underwent magnetic separation. The particles were washed multiple times in THF. A 100 mg of MNP@X, 200 mg of LitAA (0.46 mmol), 0.52 mg of *O*-ethyl-*S*-(1-methoxycarbonyl)ethyldithiocarbonate (0.0025 mmol), and 4 mL of dry tetrahydrofuran were combined in a test tube, sonicated, and purged with argon gas for half an hour. Then, 8 mg of AIBN (0.048 mmol) was introduced in two separate portions (at the begining and after twelve hours of the reaction), and the mixture was subsequently maintained at a temperature of 70 °C for twenty-four hours. The polymerization process was stopped by diluting the reaction mixture with THF and magnetic decantation. This process of redispersing and separating the mixture was executed 5 times. The particles were dispersed and stored in 50 mL THF (~2.2 mg·mL^{-1}). **MNP@PLitAA** FT IR (v_{max}, cm^{-1}): 3500-3000 (O-H), 2928 (C-H), 2863 (C-H), 1701 (C=O), 1438 (O-H), 1124 (C-N), 1038 (Si-O-Si), 538 (Fe-O).

The procedure of polymer-coated MNPs amidation with folic acid: A 10 mL of MNP@PAA/MNP@PlitAA suspension in THF underwent magnetic separation. The particles were washed multiple times in dried DMSO under the inert flow of argon gas. Folic acid was dissolved in dry DMSO (C=2.0 mg·mL^{-1}) by mixing under argon for twenty-four hours. MNP's (~20 mg), 5 mL of folic acid solution in DMSO, 4.8mg of DCC (0.024 mmol), 0.28 mg of DMAP (0.003 mmol), and 2 mL of dried DMSO were combined in a test tube, sonicated and purged with argon gas for half an hour. The particles were maintained in darkness and mixed in a shaker for forty-eight hours. Products were purified by multiple magnetic separation and redispersion processes. The particles were dispersed in 20 mL of THF.

MNP@PAA-FA FT IR (v_{max}, cm^{-1}): 3500-3000 (O-H), 2925 (C-H), 2845 (C-H), 1720 (C=O), 1597 (N-H), 1438 (O-H), 1158 (C-N), 1040 (Si-O-Si), 548 (Fe-O).

MNP@PLitAA-FA FT IR (v_{max}, cm^{-1}): 3500-3000 (O-H), 2923 (C-H), 2852 (C-H), 1720 (C=O), 1590 (N-H), 1438 (O-H), 1120 (C-N), 1032 (Si-O-Si), 550 (Fe-O).

The procedure of polymer-coated MNPs amidation with doxorubicin: A 10 mL of MNP@PAA/MNP@PLitAA suspension in THF underwent magnetic separation. The particles were washed multiple times in dried DMSO under the inert flow of argon gas. Doxorubicin hydrochloride was dissolved in dry DMSO (C=0.5 mg·mL^{-1}) under argon. MNP's (~20 mg), 5 mL of doxorubicin solution in DMSO, 4.8mg of DCC (0.024 mmol), 0.28 mg of DMAP (0.003 mmol), and 2 mL of dried DMSO were combined in a test tube, sonicated and purged with

argon gas for half an hour. The particles were maintained in darkness and mixed in a shaker for forty-eight hours. Products were purified by multiple magnetic separation and redispersion processes. The particles were dispersed in 20 mL of THF.

MNP@PAA-DOX FT IR (ν_{max}, cm^{-1}): 3500-3000 (O-H), 2929 (C-H), 1726 (C=O), 1560 (N-H), 1437 (O-H), 1252 (C-O), 1160 (C-N), 1090 (N-H), 1025 (Si-O-Si), 548 (Fe-O).

MNP@PLitAA-DOX FT IR (ν_{max}, cm^{-1}): 3500-3000 (O-H), 2927 (C-H), 2862 (C-H), 1701 (C=O), 1597 (N-H), 1438 (O-H), 1158 (C-N), 1093 (N-H), 1023 (Si-O-Si), 555 (Fe-O).

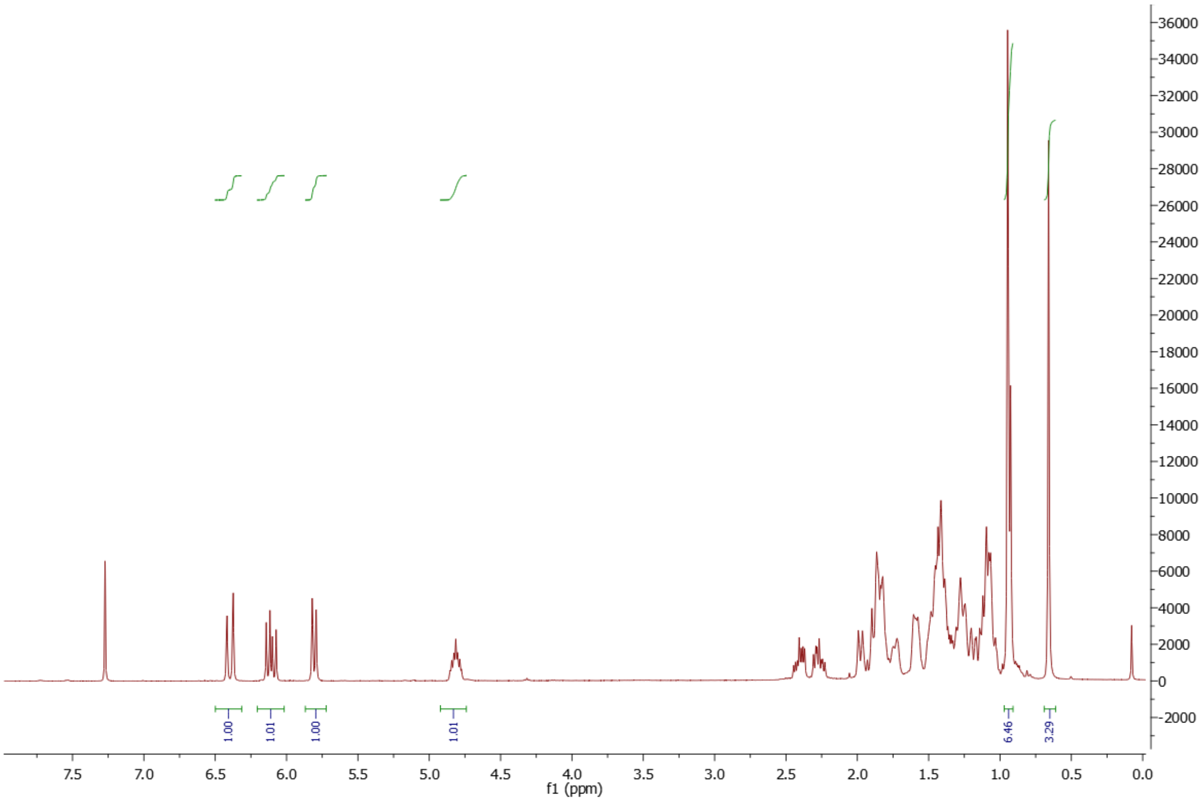

Figure S1. ^{1}H NMR spectrum of LitAA monomer.

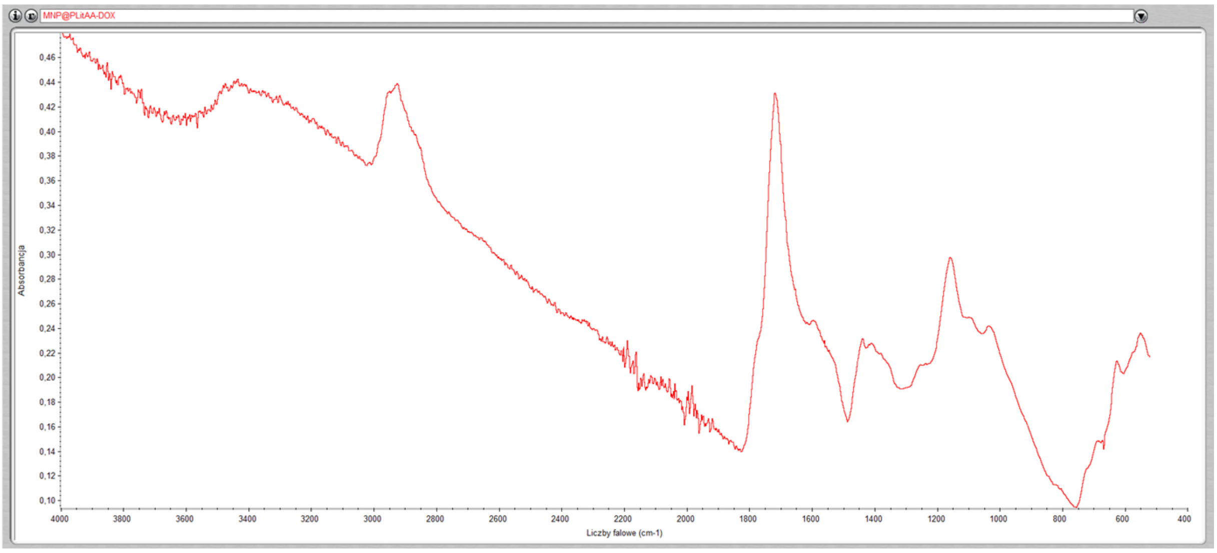

Figure S2. FT-IR spectrum of MNP@PAA-DOX.

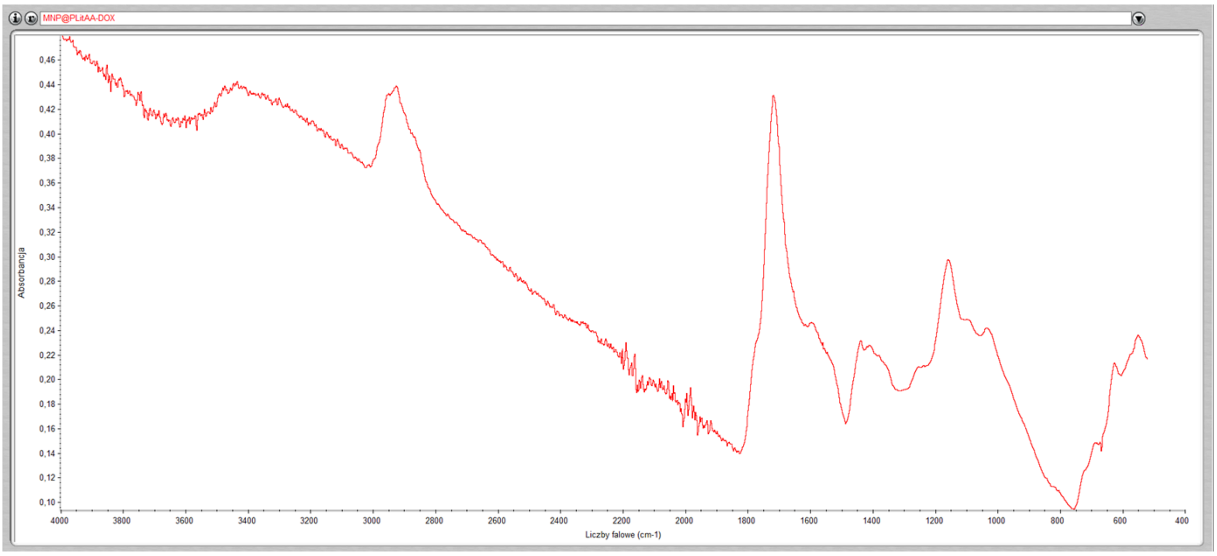

Figure S3. FT-IR spectrum of MNP@PLitAA-DOX.

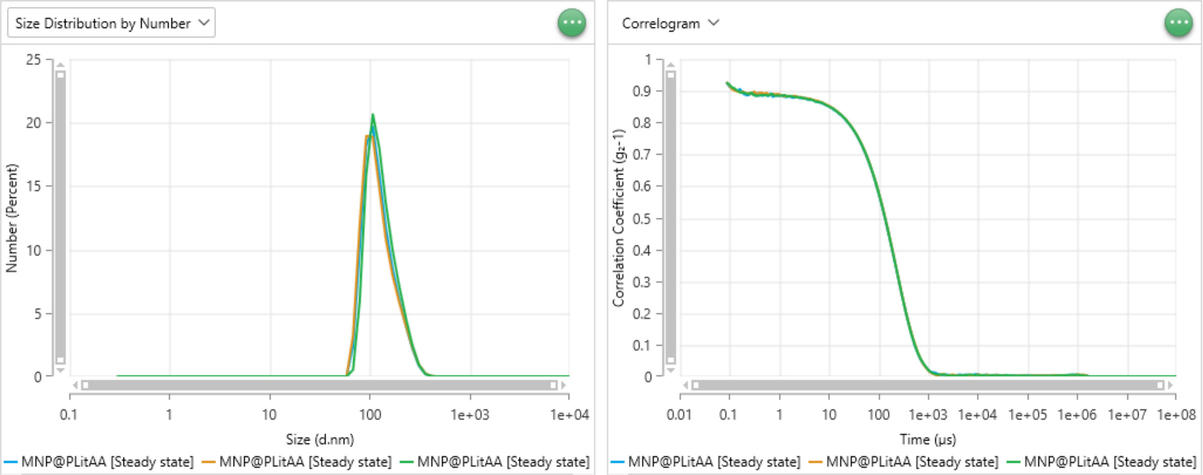

Figure S4. DLS size distribution and corellogram of MNP@PLitAA.

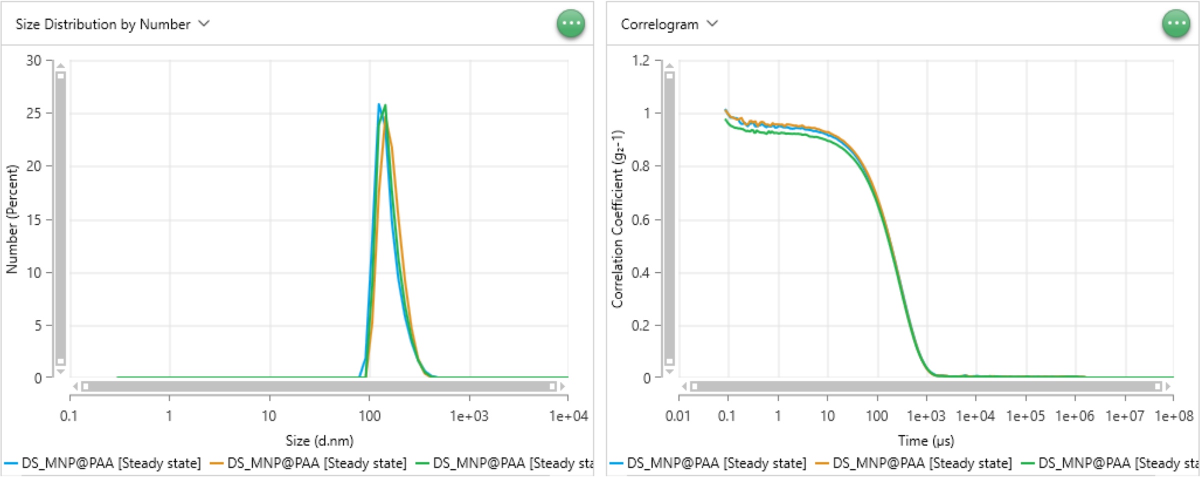

Figure S5. DLS size distribution and corellogram of MNP@PAA.

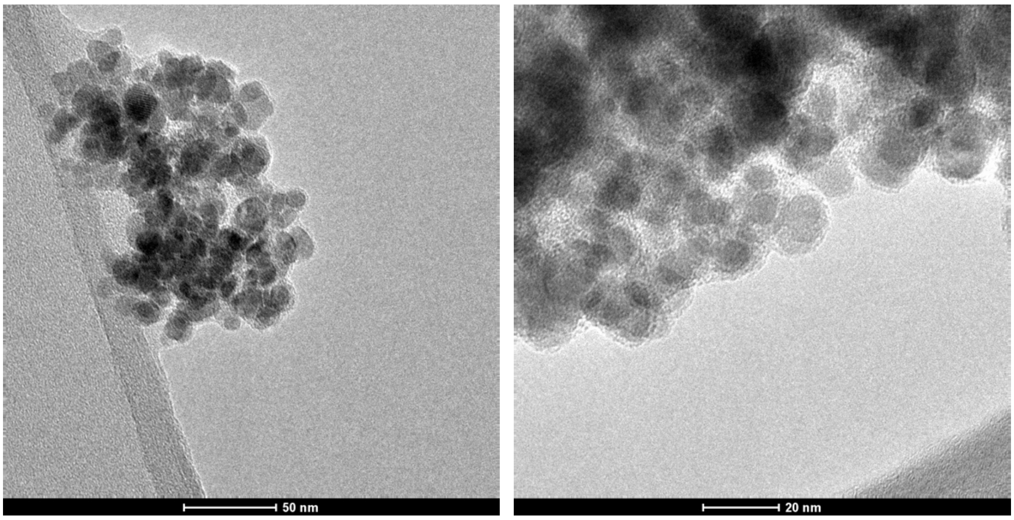

Figure S6. TEM images of MNP@PLitAA.

References

1 P. Misiak, K. Niemirowicz-Laskowska, K. H. Markiewicz, I. Misztalewska-Turkowicz, P. Wielgat, I. Kurowska, G. Siemiaszko, M. Destarac, H. Car and A. Z. Wilczewska, *Int. J. Nanomedicine*, 2020, **15**, 7263–7278.

2 K. H. Markiewicz, K. Niemirowicz-Laskowska, D. Szymczuk, K. Makarewicz, I. Misztalewska-Turkowicz, P. Wielgat, A. M. Majcher-Fitas, S. Milewska, H. Car and A. Z. Wilczewska, *Int. J. Mol. Sci.*, 2021, **22**, 4898.

3 I. Misztalewska, A. Z. Wilczewska, O. Wojtasik, K. H. Markiewicz, P. Kuchlewski and A. M. Majcher, *RSC Adv.*, 2015, **5**, 100281–100289.
